# Efficacy and Safety of Antiplatelet Agents for Adult Patients With Ischemic Moyamoya Disease

**DOI:** 10.3389/fneur.2020.608000

**Published:** 2021-01-15

**Authors:** Fei Ye, Jiaoxing Li, Tianzhu Wang, Kai Lan, Haiyan Li, Haoyuan Yin, Tongli Guo, Xiong Zhang, Tingting Yang, Jie Liang, Xiaoxin Wu, Qi Li, Wenli Sheng

**Affiliations:** ^1^Department of Neurology, The First Affiliated Hospital, Sun Yat-sen University, Guangzhou, China; ^2^Guangdong Provincial Key Laboratory of Diagnosis and Treatment of Major Neurological Diseases, The First Affiliated Hospital, Sun Yat-sen University, Guangzhou, China; ^3^Department of Neurology, The First Affiliated Hospital of Chongqing Medical University, Chongqing, China; ^4^National Health Commission Key Laboratory of Diagnosis and Treatment on Brain Functional Diseases, The First Affiliated Hospital of Chongqing Medical University, Chongqing, China; ^5^Department of Anesthesiology, Daping Hospital, Third Military Medical University (Army Medical University), Chongqing, China; ^6^Department of Anesthesiology, Troops 32268 Hospital, Dali, China; ^7^Department of Neurology, The Third Affiliated Hospital, Sun Yat-sen University, Guangzhou, China; ^8^Department of Neurosurgery, The First Affiliated Hospital of Jilin University, Changchun, China; ^9^Department of Neurology, The Affiliated Hospital of Guizhou Medical University, Guiyang, China; ^10^Department of Neurology, The Second Affiliated Hospital of Chongqing Medical University, Chongqing, China; ^11^Department of Neurology, The Third Affiliated Hospital of Chongqing Medical University, Chongqing, China

**Keywords:** Moyamoya disease, antiplatelet agents, efficacy, safety, recurrent stroke prevention

## Abstract

**Background:** The use of antiplatelet agents in ischemic moyamoya disease (MMD) is controversial. This study aimed to investigate the effectiveness and safety of antiplatelet therapy compared with conservative treatment and surgical revascularization in ischemic MMD patients.

**Methods:** Ischemic MMD patients were retrospectively enrolled from eight clinical sites from January 2013 to December 2018. Follow-up was performed through clinical visits and/or telephone interviews from first discharge to December 2019. The primary outcome was the episodes of further ischemic attacks, and the secondary outcome was the individual functional status. Risk factors for future stroke were identified by the LASSO-Cox regression model. Propensity score matching was applied to assemble a cohort of patients with similar baseline characteristics using the *TriMatch* package.

**Results:** Among 217 eligible patients, 159 patients were included in the analyses after a 1:1:1 propensity score matching. At a mean follow-up of 33 months, 12 patients (7.5%) developed further incident cerebral ischemic events (surgical:antiplatelet:conservative = 1:3:8; *p* = 0.030), 26 patients (16.4%) developed a poor functional status (surgical:antiplatelet:conservative = 7:12:7; *p* = 0.317), and 3 patients (1.8%) died of cerebral hemorrhage (surgical:antiplatelet:conservative = 1:2:0; *p* = 0.361). The survival curve showed that the risk of further cerebral ischemic attacks was lowest with surgical revascularization, while antiplatelet therapy was statistically significant for preventing recurrent risks compared with conservative treatment (χ^2^ = 8.987; *p* = 0.011). No significant difference was found in the functional status and bleeding events. The LASSO-Cox regression model revealed that a family history of MMD (HR = 6.93; 95% CI: 1.28–37.52; *p* = 0.025), a past history of stroke or transient ischemic attack (HR = 4.35; 95% CI: 1.09–17.33; *p* = 0.037), and treatment (HR = 0.05; 95% CI: 0.01–0.32; *p* = 0.001) were significantly related to the risk of recurrent strokes.

**Conclusions:** Antiplatelet agents were effective and safe in preventing further cerebral ischemic attacks in adult patients with ischemic MMD. They may be a replacement therapy for patients with surgical contraindications and for patients prior to revascularization.

## Introduction

Moyamoya disease (MMD) is an unusual cerebrovascular disease characterized by progressive steno-occlusive changes in the distal internal carotid artery (ICA) and its major branches with the development of an abnormal vascular network at the base of the brain visible on angiography (1). The Asian population has a bimodal age of presentation and is classified into two major categories: ischemic type and hemorrhagic type (2). Although current evidence has demonstrated the epidemiological and clinical characteristics of MMD, the cause of this disease is not well-known (3–8). Currently, revascularization surgery is recommended as a standard treatment for MMD patients to prevent future strokes (9). However, there are still many MMD patients who are not willing to undergo revascularization or have surgical contraindications. Consequently, it is necessary to identify a replacement therapy.

Antiplatelet agents are considered to be a vital tool in acute ischemic stroke and transient ischemic attack (TIA) management for reducing and preventing recurrent stroke risks (10, 11). To date, limited reports have focused on antiplatelet therapy for MMD, and no randomized controlled trial has suggested a lack of strongly supporting evidence indicating the benefits of this therapy. In addition, there is still controversy regarding the use of antiplatelet agents among ischemic MMD patients. Many non-Asian experts advise their use for improving microcirculation and preventing recurrent strokes, while most Asian physicians hold the opposite view, namely, that they are useless in improving blood supply and carry the potential risk of hemorrhage (12, 13). Thus, we conducted this study to evaluate the effectiveness and safety outcomes of treatment with antiplatelet agents compared with conservative treatment and surgical revascularization in ischemic MMD patients.

## Materials and Methods

### Study Design

This study was a multicenter retrospective cohort study involving adult patients with ischemic MMD who took an oral antiplatelet agent, underwent surgical revascularization, and received conservative management between January 2013 and December 2018 in eight Chinese teaching hospitals. The study was approved by the independent ethics committee of each participating teaching hospital [(2020)137]. Patient informed consent was waived due to the use of deidentified data by the clinical research review board. This study was carried out on the basis of the Strengthening the Reporting of Observational Studies in Epidemiology (STROBE) statement (14).

### Patient Selection

Patients with a clinical diagnosis of MMD were identified from the national health care system using the diagnosis-specific codes by the department of medical records in each participating hospital, including International Classification of Disease Ninth revision (ICD-9) code 437.5 and ICD-10 code I67.5. Cerebral angiography was carried out for all the patients. The diagnostic criteria for definitive or probable MMD were based on the 2012 Tokyo guideline (9, 15): (1) stenosis and/or occlusion of the terminal portion of the ICA and the proximal portion of the anterior cerebral artery (ACA) and/or middle cerebral artery (MCA), (2) abnormal vascular networks near the lesion, and (3) unilateral or bilateral involvement. Patients were eligible for inclusion in this study if they met the following criteria: (1) aged 18 or older, (2) clinical presentations of TIA or cerebral infarction, (3) no history of prior antiplatelet agents or neurosurgery, and (4) complete clinical data available. Patients who were diagnosed with moyamoya syndrome, were lost to follow-up, refused to participate, died before discharge, or had malignant tumors were excluded.

### Covariates

Electronic medical records were carefully reviewed, including hospital charts, clinic notes, operative notes, radiographic data, and therapeutic medications. All the clinical data were retrospectively collected by the data coordinators at each participating hospital in December 2019. The baseline characteristics mainly included age at symptom onset, age at diagnosis, sex, vascular risk factors (hypertension, diabetes mellitus, hyperlipidemia, active smoking, and alcohol consumption), a past history of stroke or TIA, a family history of MMD, a modified Rankin Scale (mRS) score (16), angiography findings (bilateral steno-occlusive change, Suzuki classification, and intracranial aneurysm), and clinical manifestation (TIA, lacunar infarction, and cerebral infarction). In this study, if a patient presented with an initial symptom of TIA, we did not add it to the past history of TIA. Although the RNF213 gene polymorphism and cerebral perfusion changes can influence the prognosis, these factors were excluded because they are not routine examinations in clinical practice (17, 18).

### Treatment

There are currently no clear guidelines for surgical revascularization in the treatment of MMD, but it holds a comparatively higher level of evidence than non-surgical treatment (9). Thus, randomized assignment is not applicable and unethical in clinical practice. In this study, surgical revascularization was recommended to patients with a non-emergency status, markedly ischemic symptoms, and no surgical contraindications, while non-surgical treatment was recommended to patients with mild ischemic symptoms and/or surgical contraindications. The treatment choice was independently determined by patients themselves based on their obtained medical information about the benefits and risks of different treatments. Treatments were classified into antiplatelet therapy, conservative treatment, and surgical revascularization. The treatment for patients who received antiplatelet therapy included oral aspirin 100 mg daily, oral clopidogrel 75 mg daily, and combined aspirin and clopidogrel for the first 3 weeks, followed by aspirin daily. The treatment for patients who underwent surgical revascularization procedures mainly includes direct bypass, indirect bypass, and combined bypass. Patients who had undergone surgical treatment but not surgical revascularization were excluded in this study. The conservative treatment group also included untreated participants and participants who received other medical management.

### Clinical Follow-Up

The follow-up was performed through clinical visits and/or telephone interviews from first discharge to December 2019. If patients could not be contacted in December 2019, the follow-up was determined from their last clinical visit. In this study, the primary outcome was episodes of further ischemic attacks. It was defined as an acute focal infarction of the brain or retina, including sudden onset of a new focal neurological deficit lasting 24 h or more with clinical and/or imaging evidence of infarction, sudden onset of a new focal neurological deficit lasting <24 h, or rapid worsening of an existing focal neurological deficit lasting more than 24 h but accompanied by new ischemic changes on MRI or CT of the brain (19). The secondary outcome was the individual functional status assessed by the mRS score. mRS scores ≤ 2 were regarded as a good outcome, and scores >2 were regarded as a poor outcome. The safety outcome was evaluated according to episodes of serious bleeding events based on the definition of the Global Utilization of Streptokinase and Tissue Plasminogen Activator for Occluded Coronary Arteries (GUSTO) (20). The follow-up period was defined as the time from initial discharge to the main outcome or December 2019. All reported clinical outcomes were confirmed by data coordinators who were unaware of the study-group assignments.

### Sample Size

**Figure 1:**
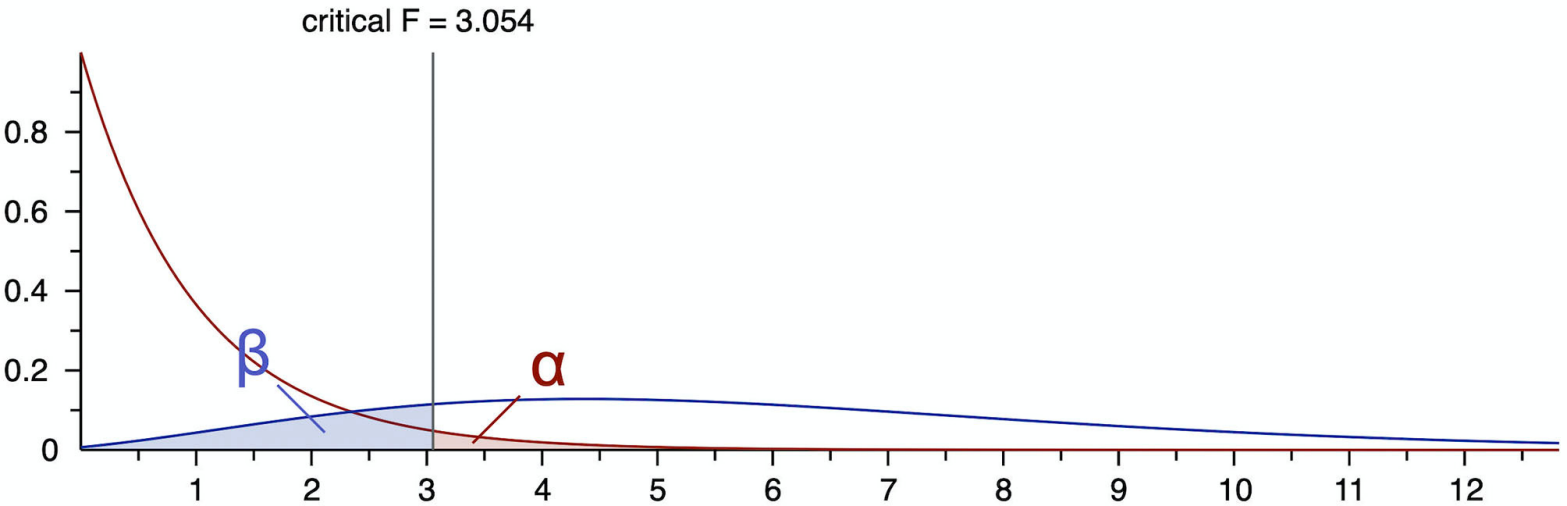
The sample size for this study based on the G*Power software.

The G^*^Power software (*version* 3.1.9.7) was applied to determine the sample size for the current study (21). Calculations were performed for three groups according to the initial treatment choice. The statistical program was set to the *F* family of tests, to a one-way analysis of variance (ANOVA), and to the “A Prior” power analysis necessary to identify sample size (22). The effect size, type I error (α), and statistical power (1 – β) were set at 0.25, 0.05, and 0.80, respectively. Based on the setup above, the total sample size was calculated to be at least 159 patients with an actual power of 0.805 (Figure 1).

### Statistical Analysis

Propensity-score matching (PSM) was widely used to adjust the between-group variations and remove the biases for non-randomized trials with two treatment alternatives (23). The propensity score (PS) is a conditional probability of receiving a particular treatment, which is calculated by a non-parsimonious multivariable logistic regression model with the treatment choice as the dependent variable and all baseline characteristics as covariates (23). In this study, a 1:1:1 matching was performed with an optimal matching algorithm and a caliper width equal to 0.25 of the standard deviation of the logit of the PS using *TriMatch* package (23, 24). In detail, the PS is estimated by logistic regression separately for all possible pairs of three groups. Each member of the antiplatelet group is matched to all surgical groups within a pre-specified caliper, and each member of the antiplatelet group is also matched to all conservative groups within the caliper. Those patients who do not have any match are removed from the sample. The PS distance is also calculated for all possible pairs between the remaining conservative group and surgical group members. The pairs with a PS distance greater than the caliper value are further eliminated. Only the unique triplets with the smallest total PS distance are finally retained in the match sample. Additionally, the standardized differences were estimated for all the baseline covariates before and after matching to assess the pre-match imbalance and post-match balance, which indicated a relatively good balance with an acceptable range of <25.0% (23).

In the matched cohort, continuous variables were reported as the median ± standard deviation. Proportions were calculated for categorical variables. ANOVA was used to analyze continuous variables. Pearson's chi-square test was used to analyze categorical variables. Univariate Cox regression analyses were performed to estimate the comparative risks of primary and secondary outcomes. Pre-specified subgroup analysis was performed on the basis of type of antiplatelet therapy (aspirin, clopidogrel, and combined antiplatelet agents). Tests for interaction were performed to assess for heterogeneity of treatment effect among subgroups. Additionally, the least absolute shrinkage and selection operator (LASSO) method was used to identify major covariates associated with future strokes from all the covariates. The major covariates were filtered via non-zero coefficients. Risk factors were then selected by multivariate Cox regression analysis using a forward stepwise method. These selected risk factors were reported as hazard ratios (HRs) with 95% confidence intervals (CIs) and *p*-values.

All statistical analyses were performed with the R Project for Statistical Computing (*version 4.0.2*) and Statistical Program for Social Science (SPSS) Statistics (*version 26*). The significance level was set at 0.05 in all tests.

## Results

### Baseline Characteristics

**Figure 2:**
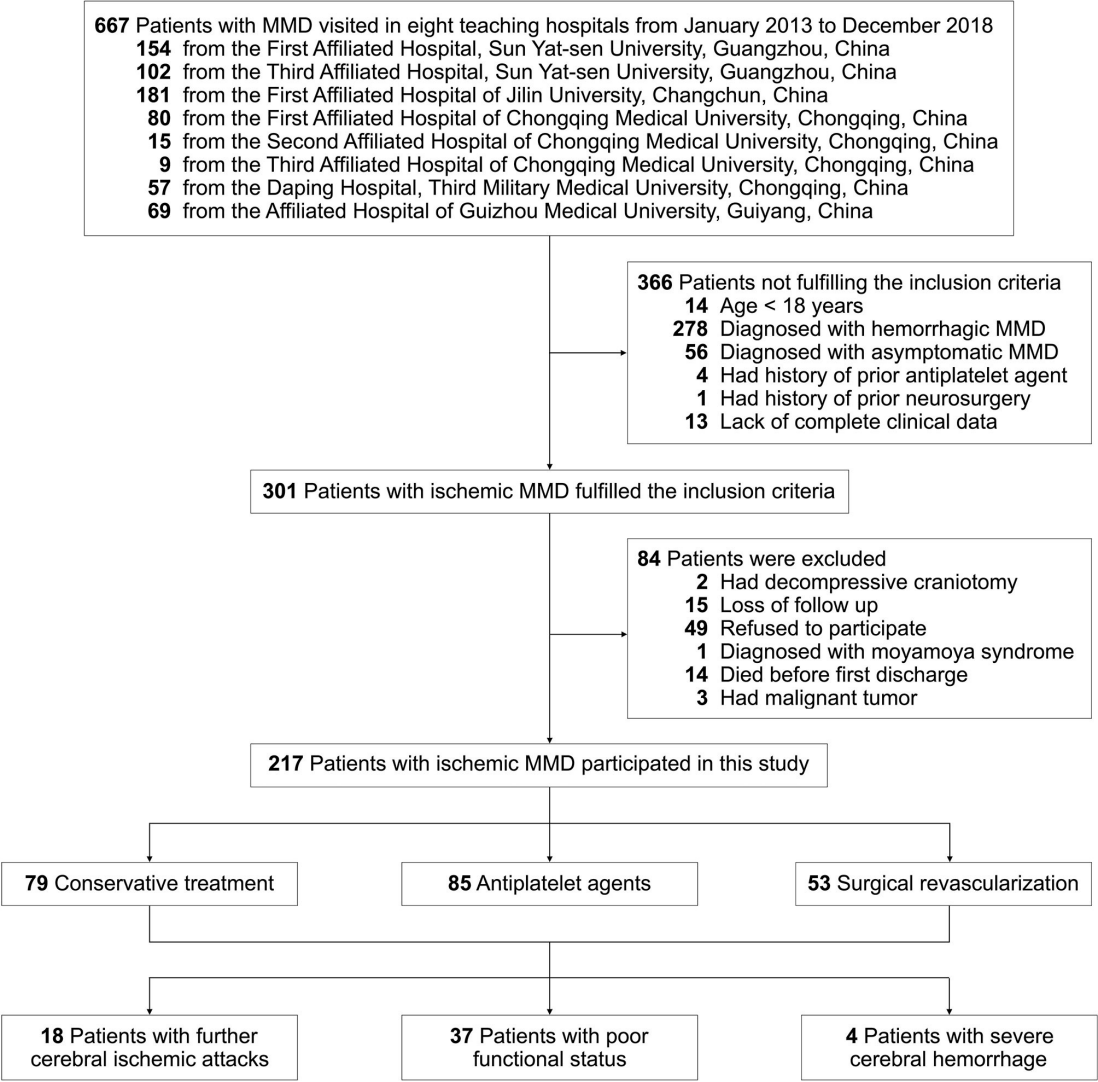
Flow chart presenting the process and outcome of this study.

This study was carried out according to the flow chart shown in Figure 2. A total of 667 MMD patients were identified at the eight Chinese teaching hospitals from January 2013 to December 2018. After applying the inclusion and exclusion criteria, 217 patients with ischemic MMD were enrolled in this study. All the baseline characteristics of these patients are summarized in Table 1. The average ages at symptom onset and diagnosis were 46 ± 12 and 47 ± 11 years, respectively. The female-to-male ratio was 0.9 (100:117). Vascular risk factors were noted in 143 patients (65.9%), including 45.6% with hypertension, 19.8% with diabetes mellitus, 7.8% with hyperlipidemia, 33.6% with active smoking, and 27.2% with alcohol consumption. The family history of MMD was approximately 5.5%. A past history of stroke or TIA was identified in 27 (12.4%) patients. The most common initial symptoms were cerebral infarction (61.3%) and TIA (32.7%). On angiography, most patients had bilateral steno-occlusive changes with Suzuki stage III–VI, but nearly 10% of them had intracranial aneurysms. Among them, 85 (39.2%) patients received antiplatelet therapy: 47 were treated with oral aspirin 100 mg daily, 18 with oral clopidogrel 75 mg daily, and 20 with combined aspirin and clopidogrel for the first 3 weeks, followed by aspirin daily. Fifty-three (24.4%) patients had undergone surgical revascularization procedures. The remaining 79 patients (36.4%) were conservatively treated.

**Table 1:** Baseline characteristics of the 217 patients with ischemic moyamoya disease.

**Characteristic**	**All (*n* = 217)**
Age at symptom onset (years)	46 ± 12
Age at diagnosis (years)	47 ± 11
Female-to-male ratio	0.9 (100:117)
**Vascular risk factors**	
Hypertension	99 (45.6%)
Diabetes mellitus	43 (19.8%)
Hyperlipidemia	17 (7.8%)
Active smoking	73 (33.6%)
Alcohol consumption	59 (27.2%)
Past history of stroke or TIA	27 (12.4%)
Family history of MMD	12 (5.5%)
**mRS score at baseline**	
0–2	179 (82.5%)
>2	38 (17.5%)
**Angiography findings**	
Bilateral	195 (89.9%)
Suzuki stage ≥III	191 (88.0%)
Intracranial aneurysm	20 (9.2%)
**Clinical manifestation**	
TIA	71 (32.7%)
Lacunar infarction	13 (6.0%)
Cerebral infarction	133 (61.3%)
**Treatment**	
Conservative treatment	79 (36.4%)
Antiplatelet therapy	85 (39.2%)
Surgical revascularization	53 (24.4%)

### Long-Term Outcomes

Of the enrolled patients, seven patients (3.2%) could not be contacted in December 2019, so the follow-up was determined from their last clinical visits. During a follow-up of 34 ± 18 months, 18 patients (8.3%) had acute ischemic strokes, 37 patients (17.1%) developed a poor functional status, and 4 patients (1.8%) died of cerebral hemorrhage (Figure 2). The median interval from first discharge to subsequent ischemic stroke was 36 ± 25 months. Eleven of the 79 (13.9%) conservatively treated patients, 6 of the 85 (7.1%) antiplatelet-treated patients, and 1 of the 53 (1.9%) surgically treated patients had future strokes (Table 2).

**Table 2:** Summary of 18 patients with recurrent ischemic stroke.

**No**	**Age at symptom onset (years)**	**Vascular risk factor**	**mRS score at baseline**	**Initial symptom**	**Outcome**			
					**Recurrent ischemic event**	**Internal (months)**	**Functional status**	**Bleeding event**
**Untreated group (*****n*** **= 11)**								
1	36–40	None	>2	Cerebral infarction	Cerebral infarction	5	Good	No
2	20–25	Hypertension	>2	Cerebral infarction	Cerebral infarction	26	Good	No
3	26–30	Hypertension	>2	Cerebral infarction	Cerebral infarction	64	Good	No
4	20–25	None	>2	Cerebral infarction	Cerebral infarction	50	Good	No
5	36–40	None	>2	Cerebral infarction	Cerebral infarction	31	Good	No
6	46–50	Hypertension	0–2	Cerebral infarction	Cerebral infarction	5	Good	No
7	30–35	Smoking, drinking	>2	Cerebral infarction	Cerebral infarction	44	Poor	No
8	46–50	None	>2	Cerebral infarction	Cerebral infarction	6	Poor	No
9	56–60	Hypertension, diabetes	0–2	Cerebral infarction	Cerebral infarction	20	Poor	No
10	50–55	None	>2	Cerebral infarction	Cerebral infarction	6	Poor	No
11	56–60	Hypertension, diabetes	0–2	Cerebral infarction	Cerebral infarction	33	Good	No
**Antiplatelet group (*****n*** **= 6)**								
1	40–45	Hypertension, hyperlipidemia	>2	Cerebral infarction	Cerebral infarction	65	Poor	Cerebral hemorrhage
2	50–55	Smoking	0–2	Cerebral infarction	Cerebral infarction	13	Poor	No
3	36–40	None	>2	Cerebral infarction	Cerebral infarction	58	Poor	Cerebral hemorrhage
4	56–60	Hypertension, diabetes, smoking	0–2	Cerebral infarction	Cerebral infarction	63	Good	No
5	30–35	Hyperlipidemia	>2	Cerebral infarction	Cerebral infarction	16	Poor	No
6	56–60	Hypertension, diabetes, drinking	>2	Cerebral infarction	Cerebral infarction	64	Poor	No
**Surgical group (*****n*** **= 1)**								
1	20–25	None	>2	Cerebral infarction	Cerebral infarction	72	Poor	No

### Development and Validation of 1:1:1 PSM

**Figure 3:**
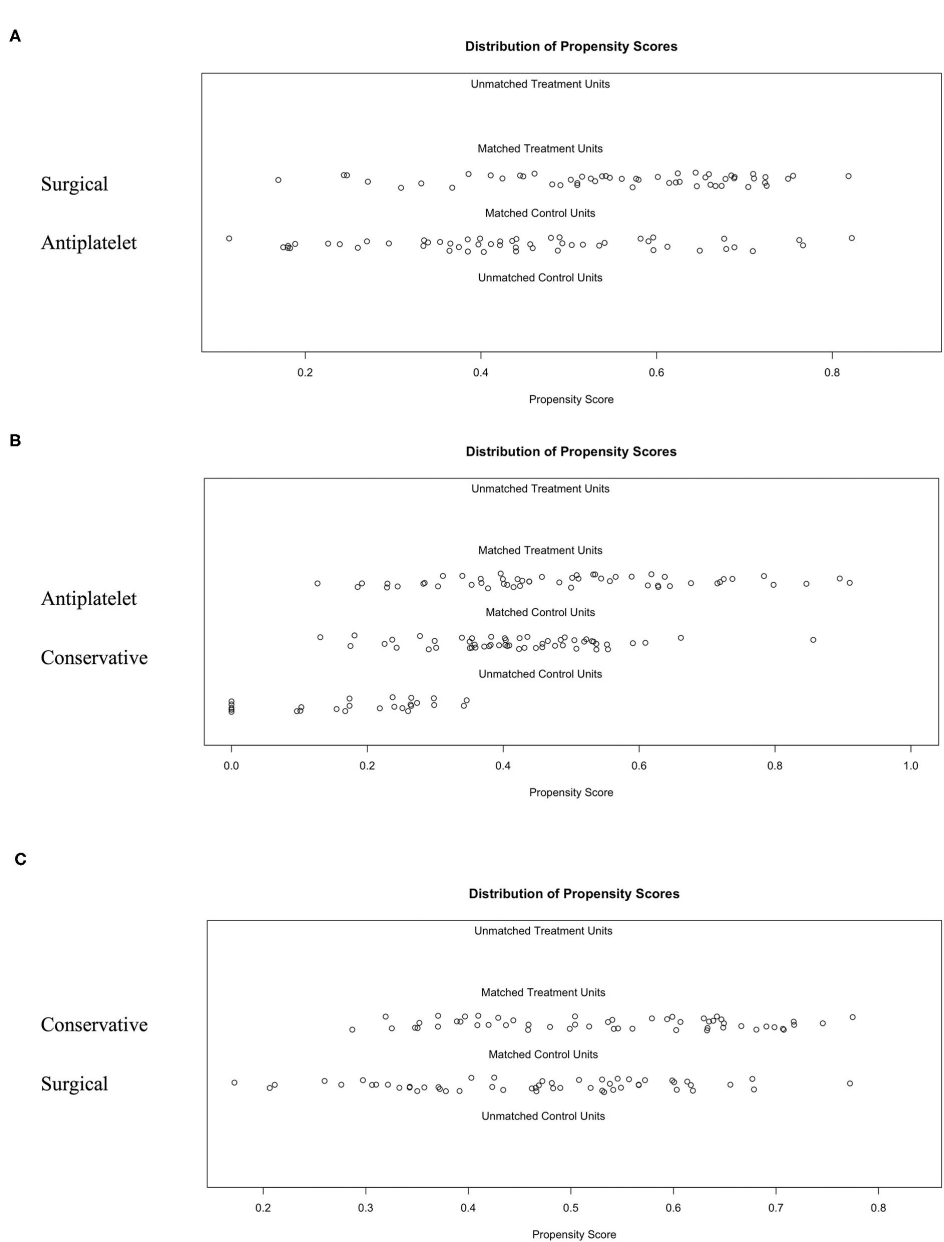
The 1:1:1 PSM results for different groups using the optimal matching algorithm and a caliper width equal to 0.25. **(A)** Surgical group matched to the antiplatelet group. **(B)** Antiplatelet group matched to the conservative group. **(C)** Identification of the accuracy between the matched conservative group and surgical group.

There were significant differences between the three groups in several of the baseline characteristics before PSM (Table 3). With the use of 1:1:1 PSM via the TriMatch package, 53 patients with antiplatelet agents were matched with 53 conservative patients and 53 patients underwent surgical revascularization (Table 3). After matching, the between-group variations were successfully removed using the optimal matching algorithm and a caliper width equal to 0.25 (Figure 3). The standardized differences were <25.0% for almost all potential risk factors, indicating only small differences between these groups (Supplementary Tables 1, 2).

**Table 3:** Comparison of clinical features and outcomes between different groups before and after matching.

**Characteristic**	**Before matching**				**After matching**			
	**Surgical (*n* = 53)**	**Antiplatelet (*n* = 85)**	**Conservative (*n* = 79)**	***p*-value**	**Surgical (*n* = 53)**	**Antiplatelet (*n* = 53)**	**Conservative (*n* = 53)**	***p*-value**
Age at symptom onset (years)	44 ± 12	48 ± 10	45 ± 13	0.064	44 ± 12	47 ± 11	47 ± 13	0.271
Age at diagnosis (years)	45 ± 12	49 ± 10	46 ± 12	0.108	45 ± 12	48 ± 11	48 ± 13	0.255
Female-to-male ratio	1.1	0.5	1.2	0.018	1.1	0.7	0.8	0.493
**Vascular risk factors**								
Hypertension	19 (35.8%)	45 (52.9%)	35 (44.3%)	0.140	19 (35.8%)	23 (43.4%)	23 (43.4%)	0.659
Diabetes mellitus	5 (9.4%)	23 (27.1%)	15 (19.0%)	0.040	5 (9.4%)	8 (15.1%)	8 (15.1%)	0.610
Hyperlipidemia	0 (0.0%)	11 (12.9%)	6 (7.6%)	0.023	0 (0.0%)	0 (0.0%)	0 (0.0%)	1.000
Active smoking	13 (24.5%)	32 (37.6%)	28 (35.4%)	0.260	13 (24.5%)	16 (30.2%)	19 (35.8%)	0.447
Alcohol consumption	12 (22.6%)	26 (30.6%)	21 (26.6%)	0.587	12 (22.6%)	13 (24.5%)	15 (28.3%)	0.792
Past history of stroke or TIA	8 (15.1%)	13 (15.3%)	6 (7.6%)	0.262	8 (15.1%)	8 (15.1%)	5 (9.4%)	0.610
Family history of MMD	4 (7.5%)	4 (4.7%)	4 (5.1%)	0.757	4 (7.5%)	2 (3.8%)	2 (3.8%)	0.591
**mRS score at baseline**								
0–2	47 (88.7%)	63 (74.1%)	69 (87.3%)	0.033	47 (88.7%)	43 (81.1%)	45 (84.9%)	0.555
>2	6 (11.3%)	22 (25.9%)	10 (12.7%)		6 (11.3%)	10 (18.9%)	8 (15.1%)	
**Angiography findings**								
Bilateral	51 (96.2%)	69 (81.2%)	75 (94.9%)	0.003	51 (96.2%)	48 (90.6%)	51 (96.2%)	0.346
Suzuki stage ≥III	46 (86.8%)	73 (85.9%)	72 (91.1%)	0.556	46 (86.8%)	46 (86.8%)	49 (92.5%)	0.569
Intracranial aneurysm	6 (11.3%)	5 (5.9%)	9 (11.4%)	0.395	6 (11.3%)	4 (7.5%)	6 (11.3%)	0.757
**Clinical manifestation**								
TIA	21 (39.6%)	23 (27.1%)	27 (34.2%)	0.273	21 (39.6%)	15 (28.3%)	15 (28.3%)	0.580
Lacunar infarction	3 (5.7%)	3 (3.5%)	7 (8.9%)		3 (5.7%)	2 (3.8%)	4 (7.5%)	
Cerebral infarction	29 (54.7%)	59 (69.4%)	45 (57.0%)		29 (54.7%)	36 (67.9%)	34 (64.2%)	
Follow-up (months)	32.2 ± 16.7	36.6 ± 20.5	33.2 ± 15.5	0.291	32.2 ± 16.7	35.6 ± 20.0	32.2 ± 14.1	0.488
**Outcomes**								
Future ischemic stroke	1 (1.9%)	6 (7.1%)	11 (13.9%)	0.042	1 (1.9%)	3 (5.7%)	8 (15.1%)	0.030
Poor functional status	7 (13.2%)	21 (24.7%)	11 (13.9%)	0.248	7 (13.2%)	12 (22.6%)	7 (13.2%)	0.317
Serious bleeding event	1 (1.9%)	3 (3.5%)	0 (0.0%)	0.244	1 (1.9%)	2 (3.8%)	0 (0.0%)	0.361

### Primary Outcome

**Figure 4:**
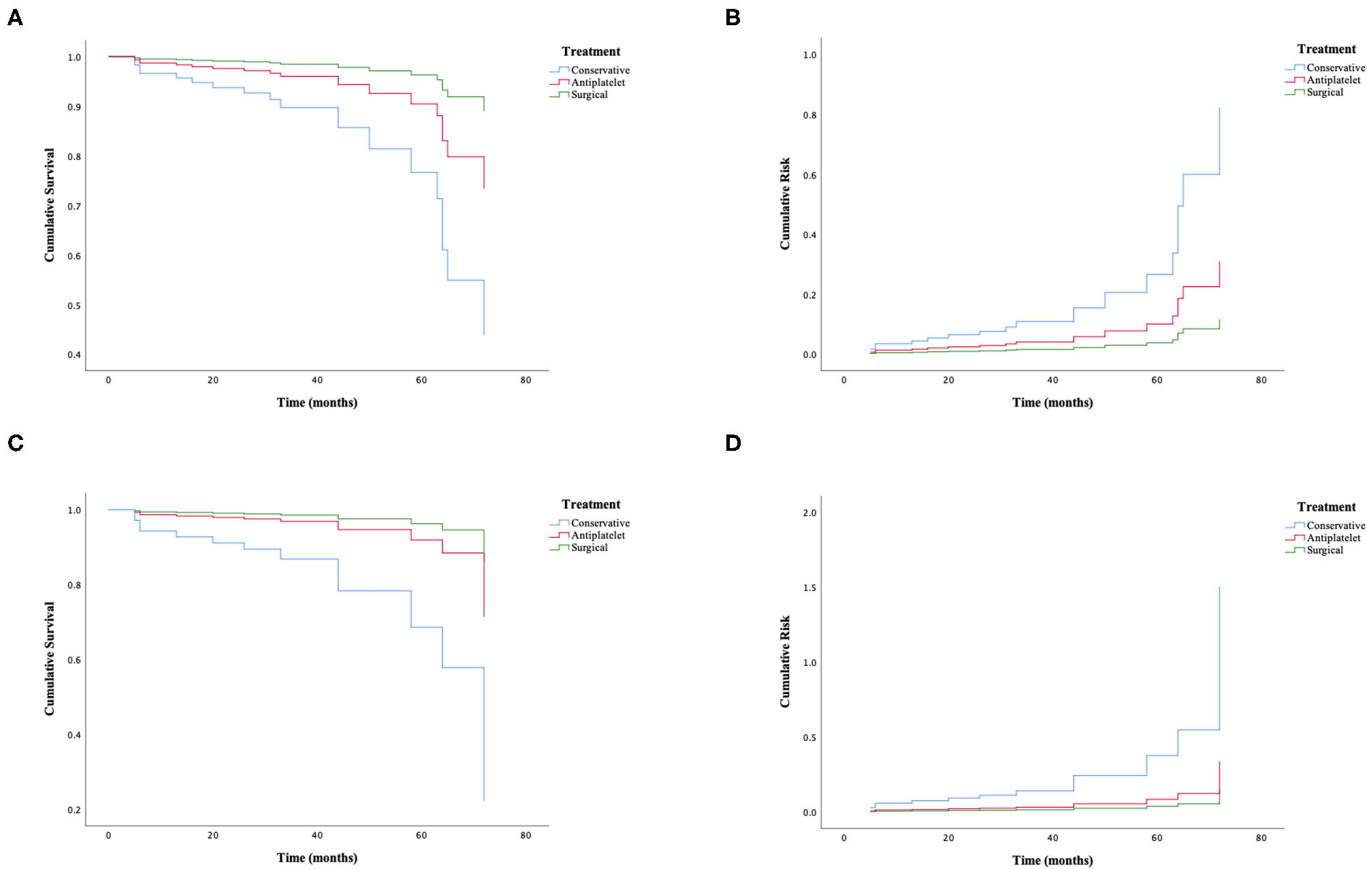
Cox regression analysis of further cerebral ischemic attacks in adult patients with ischemic moyamoya disease treated by different methods. **(A,B)** The cumulative survival before (χ^2^ = 7.188; *p* = 0.027) and after matching (χ^2^ = 8.987; *p* = 0.011). **(C,D)** The cumulative risk before (χ^2^ = 7.188; *p* = 0.027) and after matching (χ^2^ = 8.987; *p* = 0.011).

Among the matched groups, 12 patients (7.5%) developed incident ischemic stroke after an average follow-up of 33 ± 17 months. All of them had an initial symptom of cerebral infarction with a median interval period of 29 ± 25 months. The shortest interval from first discharge to the episode of cerebral infarction was 5 months, and the longest was 72 months. Of these patients, one who took antiplatelet agents died due to severe cerebral hemorrhage, and eight patients had a poor functional status. Additionally, further cerebral ischemic attack occurred in three patients (5.7%) in the antiplatelet group, compared with one patient (1.9%) in the surgical group and eight patients (15.1%) in the conservative group (*p* = 0.030). The univariate Cox regression analysis of recurrent ischemic events is shown in Figure 4, demonstrating that the probability of further cerebral ischemic attacks was lowest with surgical revascularization, while antiplatelet therapy was also statistically significant for preventing and reducing recurrent risks compared with the effects of conservative treatment. The omnibus tests of model coefficients revealed that there was a significant difference between the three risk curves (χ^2^ = 8.987; *p* = 0.011). No significant difference in recurrence risk was found between subgroups of different antiplatelet agents (*p* > 0.05; Table 4).

**Table 4:** Comparison of the clinical features and outcomes between subgroups with different antiplatelet agents.

**Characteristic**	**Aspirin (*n* = 33)**	**Clopidogrel (*n* = 10)**	**Aspirin combined with clopidogrel (*n* = 10)**	***p*-value**
Age at symptom onset (years)	47 ± 12	46 ± 10	52 ± 9	0.393
Age at diagnosis (years)	48 ± 12	46 ± 10	52 ± 10	0.463
Female-to-male ratio	0.8	0.7	0.4	0.682
**Vascular risk factors**				
Hypertension	14 (42.4%)	3 (30.0%)	6 (60.0%)	0.393
Diabetes mellitus	4 (12.1%)	1 (10.0%)	3 (30.0%)	0.339
Hyperlipidemia	0 (0.0%)	0 (0.0%)	0 (0.0%)	1.000
Active smoking	9 (27.3%)	3 (30.0%)	4 (40.0%)	0.745
Alcohol consumption	6 (18.2%)	2 (20.0%)	5 (50.0%)	0.115
Past history of stroke or TIA	7 (21.2%)	0 (0.0%)	1 (10.0%)	0.229
Family history of MMD	2 (6.1%)	0 (0.0%)	0 (0.0%)	0.533
**mRS score at baseline**				
0–2	26 (78.8%)	8 (80.0%)	9 (90.0%)	0.726
>2	7 (21.2%)	2 (20.0%)	1 (10.0%)	
**Angiography findings**				
Bilateral	31 (93.9%)	8 (80.0%)	9 (90.0%)	0.417
Suzuki stage ≥III	29 (87.9%)	8 (80.0%)	9 (90.0%)	0.769
Intracranial aneurysm	1 (3.0%)	1 (10.0%)	2 (20.0%)	0.195
**Clinical manifestation**				
TIA	8 (24.2%)	3 (30.0%)	4 (40.0%)	0.734
Lacunar infarction	2 (6.1%)	0 (0.0%)	0 (0.0%)	
Cerebral infarction	23 (69.7%)	7 (70.0%)	6 (60.0%)	
Follow-up (months)	36.9 ± 22.6	31.8 ± 17.1	35.4 ± 13.4	0.787
**Outcomes**				
Future ischemic stroke	2 (6.1%)	0 (0.0%)	1 (10.0%)	0.618
Poor functional status	8 (24.2%)	3 (30.0%)	1 (10.0%)	0.530
Serious bleeding event	2 (6.1%)	0 (0.0%)	0 (0.0%)	0.533

### Secondary Outcome

In the matched groups, 26 patients (16.4%) had a poor functional status at follow-up. Of these patients, 12 (46.2%) had moderate-to-severe neurological dysfunction at baseline, and these patients accounted for a greater proportion than the 24 patients (15.1%) among all subjects. In the present study, a poor functional status occurred in 12 patients (22.6%) in the antiplatelet group, compared with 7 patients (13.2%) in the surgical group and 7 patients (13.2%) in the conservative group (Table 3). Although antiplatelet therapy was found to be associated with a comparatively higher risk of a poor outcome, the univariate Cox regression analysis showed no significant difference between groups (χ^2^ = 0.400; *p* = 0.819).

### Safety Outcome

In the matched groups, three deaths (1.8%) occurred because of severe cerebral hemorrhages, including two deaths among patients who were treated with antiplatelet agents and one patient who was treated with surgical revascularization (Table 3). No hemorrhagic events were observed in the conservative groups. No significant difference was observed among groups and subgroups, but antiplatelet therapy showed a relatively higher risk of bleeding (3.8%) in two patients treated with aspirin (Table 4).

### Risk Factors for Further Cerebral Ischemic Attacks

**Figure 5:**
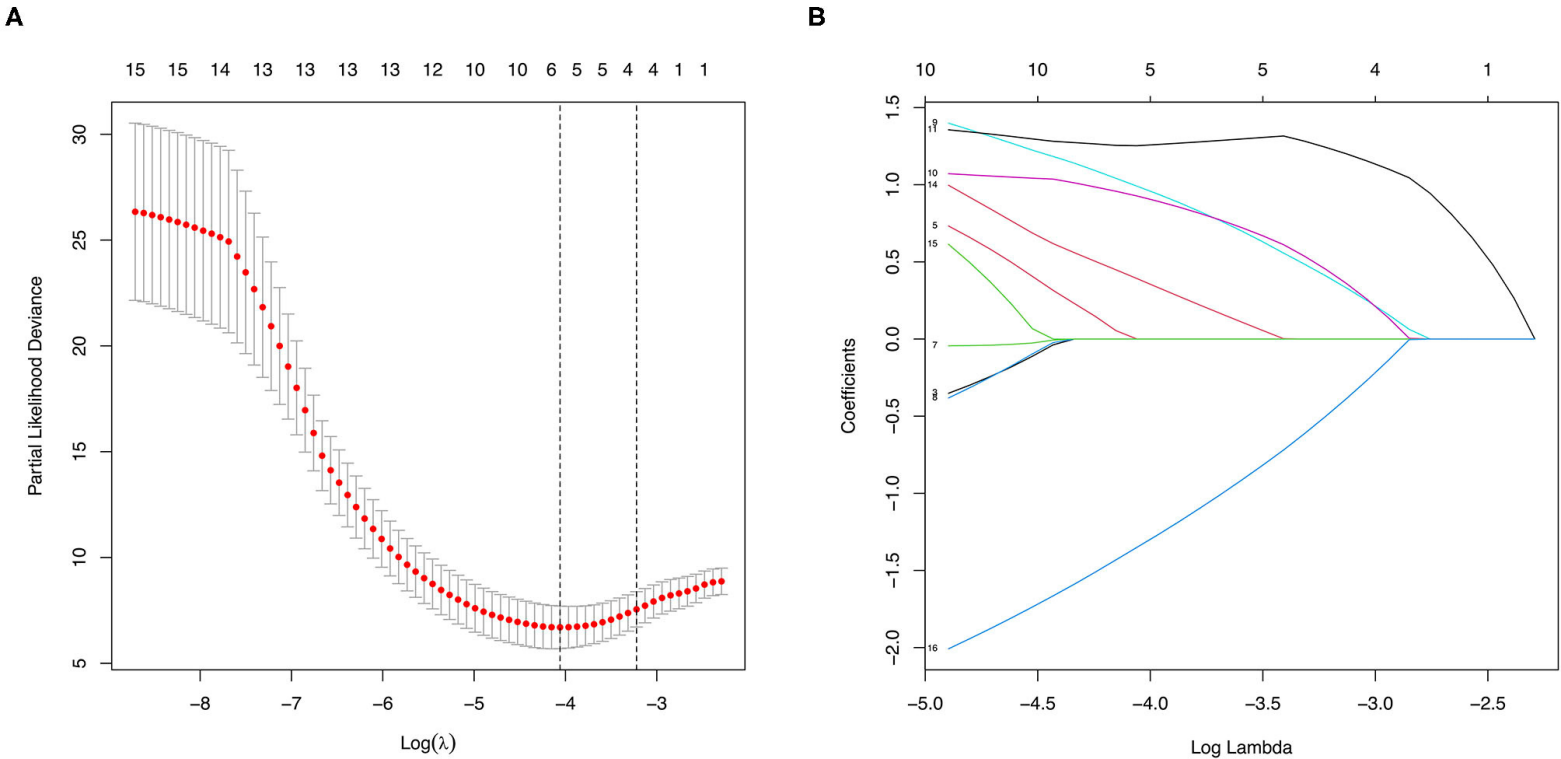
Features selection using the least absolute shrinkage and selection operator (LASSO) method. **(A)** Tuning parameter (λ) selection in the LASSO model used five-fold cross-validation via minimum criteria. The partial likelihood deviance (binomial deviance) curve was plotted vs. log(λ). Dotted vertical lines were drawn at the optimal values by using the minimum criteria and the 1 standard error of the minimum criteria (the 1-SE criteria). **(B)** LASSO coefficient profiles of the 16 covariates. A coefficient profile plot was produced against the log (λ) sequence. Vertical line was drawn at the value selected using five-fold cross-validation, where optimal λ resulted in 5 non-zero coefficients.

All the baseline characteristics and treatment choices were entered as covariates. Five potential major factors with non-zero coefficients were selected from the 16 clinical features in terms of the 159 patients via the LASSO regression model (1:3 ratio, Figure 5), including a family history of MMD, a past history of stroke or TIA, an mRS score at baseline, clinical manifestation, and treatment choice. A family history of MMD (HR = 6.93; 95% CI: 1.28–37.52; *p* = 0.025), a past history of stroke or TIA (HR = 4.35; 95% CI: 1.09–17.33; *p* = 0.037), and treatment (HR = 0.05; 95% CI: 0.01–0.32; *p* = 0.001) were identified as risk factors in multivariate Cox regression analysis using the forward stepwise method. No significant correlations were observed between further cerebral ischemic attacks and other factors (*p* > 0.05).

## Discussion

In the present study, we evaluated whether antiplatelet agents showed a potential effect in adult patients with ischemic MMD. To clarify the efficacy and safety of antiplatelet agents in ischemic MMD, we performed this retrospective cohort study and assessed the outcomes of 217 patients during a follow-up of 34 ± 18 months; the participants included 79 conservatively treated patients, 85 patients treated with antiplatelet agents, and 53 patients treated with surgical revascularization. Our results showed that antiplatelet agents were effective in preventing and reducing further cerebral ischemic attacks in these patients.

The baseline characteristics were similar to those of samples in previous studies, and the ratio of women to men (0.9) and family history of MMD (5.5%) in this study appeared to be similar to the respective parameters in the study by Duan et al. (6), which indicated an important influence of heredity. Most of the patients were diagnosed with bilateral steno-occlusive changes (89.9%), and a few were diagnosed with intracranial aneurysms (9.2%) on angiography, a finding that was consistent with a previous study by Kraemer et al. (25) and remained unclear. In addition, as Hervé et al. (26) found, the most common symptom was cerebral infarction (61.3%), followed by TIA (32.7%). However, the average ages at symptom onset (46 ± 12 years) and diagnosis (47 ± 11 years) in our study were comparably older than those in previous studies (3–8) because our strict criteria excluded a large portion of pediatric patients and adult patients with hemorrhagic MMD, which may explain the different results. Furthermore, our study revealed that a family history of MMD, a past history of stroke or TIA, and treatment choice could be independent risk factors for additional cerebral ischemic events based on a LASSO-Cox regression model.

Similarly, the univariate Cox regression analysis showed that revascularization surgery performed best for the treatment of ischemic MMD, with the lowest rate of further cerebral ischemic events (1.9%) and longest interval period between strokes (72 months) in this study, which was generally employed as the first choice to treat MMD patients to prevent future strokes with strong evidence (9, 27, 28). Interestingly, our study also showed that compared with conservative treatment, antiplatelet therapy was beneficial for reducing further cerebral ischemic events (5.7 vs. 15.1%) and extending the interval period (45 months vs. 18 months) among these patients. It was obvious that conservative treatment was inferior to surgical revascularization with regard to the prognosis of MMD patients. In the past, observational studies categorized antiplatelet therapy as conservative treatment for statistical analysis. Thus, the effectiveness of antiplatelet agents could be weakened due to the mixture of no treatment and treatment with other medications. In addition, current studies suggested that aspirin was effective in the postoperative management of ischemic MMD (29, 30). However, the use of antiplatelet agents in the treatment of MMD is controversial, and few studies have reported the efficacy of antiplatelet agents among non-surgical patients or before surgery. The majority of non-Asian experts advised using them for improving microcirculation and preventing recurrent strokes, while most Asian physicians held the opposite view that they were useless in improving blood supply and carried the potential risk of hemorrhages (12, 13). A recent study in Japan demonstrated that antiplatelet agents were effective in improving cerebral perfusion in adult patients with symptomatically ischemic MMD (31, 32). Likewise, our study focused on the efficacy and safety of antiplatelet agents used for adult MMD patients with the initial symptom of ischemia and compared their efficacy and safety with those of conservative treatment and surgical revascularization. Regarding the primary outcome, the recurrence rate of cerebral ischemic attack in the antiplatelet group was 5.7%, 1.9% in the surgical group, and 15.1% in the conservative group (*p* = 0.030), indicating that antiplatelet treatment could be effective for preventing ischemic stroke or TIA. However, regarding the secondary outcome, the patients in the antiplatelet group account for a higher proportion of poor neurological function than the other two groups, although statistical significance was absent among them. We noticed that the number of patients with mRS > 2 at baseline in the antiplatelet group was also higher than that in the other two groups. Therefore, we could not conclude that antiplatelet agents are responsible for a poorer functional status. We re-analyzed these 12 patients with recurrent strokes. Among these patients, four patients presented with a baseline mRS of 0–2 (surgical:antiplatelet:conservative = 0:1:3), suggesting a trend to benefit these patients with mild symptoms at baseline from both surgical revascularization and antiplatelet therapy could be compared with conservative treatment. However, because of our small sample size, we still needed a large sample size randomized control study to prove this finding. Thus, these differences may influence the secondary outcome. Because of the limitation of the retrospective study, some covariates could not be adjusted by the propensity score matching method. Moreover, the insufficient follow-up period weakened the statistical power in the secondary outcome comparison. Although the mechanism of MMD is still unclear, pathological studies have demonstrated that endovascular thrombosis and hyperplasia of smooth muscle cells lead to the progressive stenosis or occlusion of the distal ICA, which could explain the cause of ischemic symptoms (1). In addition, antiplatelet agents are widely used for the primary or secondary preventive management of acute ischemic stroke and TIA patients (10, 11). Aspirin inhibits the metabolism of arachidonic acid to prostacyclin 2 (PGI2) and serum thromboxane A2 (TXA2) by blocking cyclooxygenase-1 (COX-1) to reduce thrombosis. Clopidogrel is an adenosine diphosphate (ADP) receptor inhibitor that selectively interacts with P2Y12 receptor to depress platelet aggregation. Indeed, antiplatelet agents have been effectively applied to prevent further cerebral ischemic events because of their ability to improve the development of luminal thrombosis.

There were also several limitations of this study. First, this study is a non-randomized observational study with a small sample size. Second, although PSM is used to correct baseline differences, selection biases may not be removed because our patients were enrolled from eight clinical sites. Professional performances at different clinical centers could also affect the validity of the conclusions. Third, our subjects were all Asians; thus, whether our results are applicable to Western populations is unknown. Finally, genetic factors were not assessed in our study. Thus, a large randomized controlled trial is necessary.

In brief, we found that surgical revascularization was the first treatment choice, but antiplatelet therapy was a relatively significant management strategy for adult patients with ischemic MMD for reducing and preventing future strokes. Therefore, antiplatelet therapy may be a replacement therapy for patients with surgical contraindications and for patients before revascularization. We believe that this information about actual therapeutic practices in China will be helpful for the management of ischemic MMD.

## Data Availability Statement

The original contributions presented in the study are included in the article/Supplementary Materials, further inquiries can be directed to the corresponding author/s.

## Ethics Statement

The studies involving human participants were reviewed and approved by the independent ethics committe of the First Affiliated Hospital of Sun Yat-sen University and each participating medical center's ethics committee. Written informed consent for participation was not required for this study in accordance with the national legislation and the institutional requirements.

## Author Contributions

FY designed the study, drafted the manuscript, and contributed to the discussion. JLi, TW, and KL designed the study, collected the data, and analyzed the data. HL, TG, XZ, and TY collected the data and contributed to the discussion. JLia and XW collected the data. QL and WS designed the study, reviewed the manuscript, and contributed to the discussion. All authors contributed to the article and approved the submitted version.

## Conflict of Interest

The authors declare that the research was conducted in the absence of any commercial or financial relationships that could be construed as a potential conflict of interest.
